# Engineering of vitamin prototrophy in *Clostridium ljungdahlii* and *Clostridium autoethanogenum*

**DOI:** 10.1007/s00253-019-09763-6

**Published:** 2019-04-10

**Authors:** Florence J. Annan, Bakir Al-Sinawi, Christopher M. Humphreys, Rupert Norman, Klaus Winzer, Michael Köpke, Sean D. Simpson, Nigel P. Minton, Anne M. Henstra

**Affiliations:** 1 BBSRC/EPSRC Synthetic Biology Research Centre, School of Life Sciences, University of Nottingham, Nottingham, NG7 2RD UK; 2 University of New-South Wales (UNSW) Sydney, Kensington, Australia; 3 LanzaTech Inc., 8045 Lamon Avenue, Suite 400, Skokie, IL USA

**Keywords:** Biosynthetic pathway, Allele coupled exchange, Pantothenate, Thiamine, Biotin, Gas fermentation

## Abstract

*Clostridium autoethanogenum* and *Clostridium ljungdahlii* are physiologically and genetically very similar strict anaerobic acetogens capable of growth on carbon monoxide as sole carbon source. While exact nutritional requirements have not been reported, we observed that for growth, the addition of vitamins to media already containing yeast extract was required, an indication that these are fastidious microorganisms. Elimination of complex components and individual vitamins from the medium revealed that the only organic compounds required for growth were pantothenate, biotin and thiamine. Analysis of the genome sequences revealed that three genes were missing from pantothenate and thiamine biosynthetic pathways, and five genes were absent from the pathway for biotin biosynthesis. Prototrophy in *C. autoethanogenum* and *C. ljungdahlii* for pantothenate was obtained by the introduction of plasmids carrying the heterologous gene clusters *panBCD* from *Clostridium acetobutylicum*, and for thiamine by the introduction of the *thiC-purF* operon from *Clostridium ragsdalei*. Integration of *panBCD* into the chromosome through allele-coupled exchange also conveyed prototrophy. *C. autoethanogenum* was converted to biotin prototrophy with gene sets *bioBDF* and *bioHCA* from *Desulfotomaculum nigrificans* strain CO-1-SRB, on plasmid and integrated in the chromosome. The genes could be used as auxotrophic selection markers in recombinant DNA technology. Additionally, transformation with a subset of the genes for pantothenate biosynthesis extended selection options with the pantothenate precursors pantolactone and/or beta-alanine. Similarly, growth was obtained with the biotin precursor pimelate combined with genes *bioYDA* from *C. acetobutylicum*. The work raises questions whether alternative steps exist in biotin and thiamine biosynthesis pathways in these acetogens.

**Electronic supplementary material:**

The online version of this article (10.1007/s00253-019-09763-6) contains supplementary material, which is available to authorized users.

## Introduction

Carboxydotrophic acetogenic *Clostridium* spp. are well placed to play important roles in carbon capture and production of fuels and platform chemicals from various wastes and renewable resources by synthesis gas fermentation (Marcellin et al. 2016; Liew et al. 2016; Dürre 2017; Humphreys and Minton 2018). The best studied members of this group are the closely related *Clostridium autoethanogenum* and *Clostridium ljungdahlii*, and genome scale metabolic models are available for these strains (Bruno-Barcena et al. 2013; Marcellin et al. 2016; Nagarajan et al. 2013). Many *Clostridium* spp. are fastidious microorganisms and are by default grown in complex media. This is also true for *C. autoethanogenum* and *C. ljungdahlii*. Use of defined media avoids interference of unspecified organic compounds present in complex media, potentially reduces cost of the medium, helps to reduce risk of infection of cultures and improves process exergy (Dadak et al. 2016; Aghbashlo et al. 2016). Fastidious microorganisms require the addition of organic molecules for which they are auxotrophic to support their growth. These are often specific amino acids or vitamins, for which their biosynthesis pathway is incomplete. Heterologous genes for absent metabolic steps can be used as selectable markers to ensure the stable maintenance of recombinant plasmids carrying a desirable trait. Traditionally, such plasmids are maintained through incorporation into the plasmid of genes that confer resistance against antibiotics or other toxic compounds and the addition of these toxic compounds to the growth media. The presence of antibiotics can affect cellular function, while their cost can preclude their use in large-scale processes. Moreover, in an era of increasing antimicrobial resistance, the use of such genes should be avoided (Pronk 2002).

Genetic tools are available for markerless integration of heterologous genes into the genomes of various *Clostridium* spp. (Minton et al. 2016)*.* This creates the opportunity to develop stable prototrophic strains of fastidious microorganisms once it is known which genes are needed through their integration into the chromosome. Of particular utility is the method allele-coupled exchange (ACE), which can be used to introduce DNA cargo of any size or complexity at defined genomic loci, most commonly *pyrE*, a pivotal gene involved in uracil biosynthesis (Heap et al. 2012). The selection used is dependent whether the targeted pyrE allele is mutant or wild type. In the most useful configuration, the former is employed and the desired payload is integrated into the chromosome concomitant with restoration of the cell to uracil prototrophy, a readily selectable phenotype. The use of ACE to both create *pyrE* deletions and thereafter correct back to wild type was recently demonstrated in *C. autoethanogenum* using *pyrE* (Liew et al. 2017). Insertion of genes into the chromosomes of *C. autoethanogenum* or *C. ljungdahlii* by ACE is yet to be demonstrated.

Here, we investigate auxotrophy in *C. autoethanogenum* and *C. ljungdahlii*, identify the genes that are needed to create prototrophy for pantothenate, biotin and thiamin and transform these microorganisms with heterologous genes to prototrophy. Removing auxotrophies will allow growth on completely defined media without any other carbon sources other than gas/sugar and benefit systems biology studies. Also, removal of vitamin supplementation would lower process costs as growth medium makes up a significant part of costs of an industrial process. The ability of *C. autoethanogenum* transformed with the *bioYDE* gene cluster of *C. acetobutylicum* to grow when pimelic acid is added to the medium suggests that unknown pathway steps exist for BioW and BioF in biotin synthesis. Similarly, the apparent lack of *thiG* and *thiH* did not prevent growth of strains transformed with the *thiC-purF* operon from *Clostridium ragsdalei* in the absence of thiamine and suggests the presence of alternatives. Additionally, the reliance on *panB*, *panC* and *panD* and low availability of pantothenate in yeast extract allow multifactorial or iterative plasmid-based genetic engineering in combination with the use of pantolactone and beta-alanine as a tool.

## Materials and methods

### Strains

*Clostridium autoethanogenum* DSM10061 (Abrini et al. 1994), *Clostridium ljungdahlii* DSM13528 (Tanner et al. 1993) and *Clostridium ragsdalei* DSM15248 were obtained from the Deutsche Sammlung von Mikroorganismen und Zellkulturen (DSMZ, Germany). *Clostridium acetobutylicum* ATCC 824 was kindly provided by H. Bahl (University of Rostock, Germany). *E. coli* CA434 (Purdy et al. 2002) was kindly provided by M. Young (University College Wales, Aberystwyth, UK).

### Growth conditions

Bacterial stocks were kept at − 80 °C in medium with 15% *v*/*v* DMSO or 25% *v*/*v* glycerol as cryoprotectant and routinely (pre-)grown at 37 °C in YTF medium (composition below) or for *E. coli* in LB medium (in g/L: yeast extract (YE), 5; tryptone, 10; NaCl, 10). Solid media contained 15 g/L bacteriological agar (Oxoid) in addition to the components of the liquid medium. Cultures of *Clostridium* strains were grown anaerobically in a MG1000 workstation equipped with TG airlock (Don Whitley Scientific, UK) supplied with N_2_, CO_2_ and H_2_ or a PureLab glovebox system (Inert, Amesbury, MA, USA) or when CO was used, in glass serum bottles that were sealed with butyl rubber stopper (Rubber B.V., Netherlands) inside the anaerobic workstation. A complex medium (YTF medium), or a defined 2-(N-morpholino)-ethanesulfonic acid (MES) buffered mineral medium (MBM medium) with vitamins, was used. YTF medium contained per litre: yeast extract, 10 g; tryptone, 16 g; fructose, 10 g; sodium chloride, 0.2 g; and 1 mL vitamin solution (see below). MBM medium, after Rajagopalan et al. (2002), contained (per L) 5.0 g MES, pH 5.8; 25 mL macronutrient solution (MNS); 10 mL trace element solution (TES); and 1 mL vitamin solution. MNS contained (in g/L) NH_4_Cl, 40; KH_2_PO_4_, 8.0; MgSO_4_·7H_2_O, 8.0; KCl, 4.0; CaCl_2_, 0.8; and NaCl, 32. TES contained (in g/L) nitrilotriacetic acid, 2; MnSO_4_·H_2_O, 1; Fe(SO_4_)_2_(NH_4_)_2_·6H_2_O, 0.8; CoCl_2_·6H_2_O, 0.2; ZnSO_4_·7H_2_O, 0.02; CuCl_2_·2H_2_O, 0.02; NaMoO_4_·2H_2_O, 0.02; Na_2_SeO_3_, 0.02; NiCl_2_·6H_2_O, 0.02; and Na_2_WO_4_·2H_2_O, 0.02. The vitamin solution contained (in mg/L) thiamine HCl, 50; riboflavin, 50; nicotinic acid, 50; calcium pantothenate, 50; pyridoxine-HCl, 10; biotin, 20; folic acid, 20; 4-aminobenzoic acid, 50; cyanocobalamin, 50; and lipoic acid, 50. Fructose (40 mM) was used as growth carbon and energy source, except when CO (P_CO_ = 100 kPa) was used, which was added to the existing serum bottle gas phase. L-cysteine (1 g/L) served as reducing agent and source of sulfur, except when sodium sulfide (2.5 mM) was used. The following supplements were added to or omitted from MBM medium as indicated: YE (10 g/L), vitamin-free casein acid hydrolysate (CAS, 10 g/L) (Sigma), 5-fluoroorotic acid (FOA, 500 mg/L), uracil (U, 1 mg/L), pantolactone (50 μg/L), beta-alanine (50 μg/L) and pimelic acid (20 μg/L). Antibiotics were used when indicated, at following concentrations: clarithromycin (Cl), 6 mg/L; thiamphenicol (Tm), 7.5 mg/L; erythromycin (Em), 500 mg/L; chloramphenicol (Cm), 25 mg/L.

### Phenotypic analysis

Growth of *C. ljungdahlii* and *C. autoethanogenum* in medium lacking one or more sources of amino acids or vitamins was tested as indicated (Table 1) to identify essential amino acids and vitamins needed for growth. The test was carried out in 25-mL Hungate tubes that contained 20 mL of medium with inoculation volume of 1% *v*/*v* over 5 serial transfers and 48 h between serial transfers to allow the culture to grow. The optical densities (OD) of cultures in the Hungate tubes were recorded using a biowave CO8000 portable cell density meter (Biochrom, UK). Qualitative growth was indicated by an increase in OD and confirmed by presence of cells of the correct morphology in the fifth dilution by phase contrast microscopy (Nikon Eclipse Ci). The initial inoculum for the serial transfer was a culture in exponential growth phase, grown in MBM + CAS (MBM with CAS added), cysteine and full vitamin complement added. Requirement of amino acids for growth was tested in MBM medium with sodium sulfide and full vitamin complement added. Requirement of vitamins for growth was tested in MBM medium supplemented with CAS, cysteine and a vitamin solution lacking one of each of the vitamins.

**Table 1:** Vitamin requirements of wild-type *C. autoethanogenum* and *C. ljungdahlii* strains and of derivative strains with pathways for biotin or pantothenate biosynthesis complemented by integration of genes in the chromosome, or both by genes carried on plasmid

Strain	Medium																
	YTF		MBM + V^3^ and indicated compound omitted.											MBM			
		+V^3^	- L-cysteine	- 4-aminobenzoic acid	- cyanocobalamin	- riboflavin	- lipoic acid	- pantothenic acid	- biotin	- thiamine	- nicotinic acid	- pyridoxin-HCl	- folic acid	+ PBT^3^	+ BT^3^	+ PT^3^	+ T^3^
wt^a^	−	+	+	+	+	+	+	–	–	–	+	+	+	+	–	–	–
PANTS^a^	+	+	+											+	+	–	–
BIO^b^	–	+	+											+	–	+	–
biopants^b^	+	+	+											+	+	+	+

^a^*C. autoethanogenum* and *C. ljungdahlii*

^b^*C. autoethanogenum* only

*V* all vitamins, *P* pantothenate, *B* biotin, *T* thiamine

### Genotypic analysis

Completeness of metabolic pathways for vitamin biosynthesis in *C. autoethanogenum* and *C. ljungdahlii* was assessed using the published whole genome metabolic models iHN637 and iCLAU786 (Nagarajan et al. 2013; Marcellin et al. 2016) and KEGG pathway database information (Kanehisa et al. 2017). Sequences to validate identified genes, or of orthologs of missing genes in incomplete pathways, were obtained from KEGG or UNIPROT databases (Bateman et al. 2017) and used in BLAST (Altschul et al. 1990) provided by NCBI (Agarwala et al. 2017). Whole genome sequences of *C. autoethanogenum* with GenBank accession numbers GCA_000484505.1, GCA_001484725 and *C. ljungdahlii* GCA_000143685.1 were used (Brown et al. 2014; Humphreys et al. 2015; Köpke et al. 2010).

### Molecular genetic tools

All used enzymes were from New England Biolabs (NEB), gel extraction and plasmid miniprep kits were from Qiagen, Zymo Research or NEB, and the manufacturer’s protocols were followed. PCR primers were synthesised by Sigma, Eurofins Genomics or LifeTechnologies (Table 2).

**Table 2:** Primers used in this study

Primer ID	Sequence	Endonuclease/template
Pantothenate		
panBfwd	tatacatatgaaaaataccactgaaac	*Nde*I/*C. acetobutylicum*
panCfwd	tatacatatggaaattttgcattcc	*Nde*I/*C. acetobutylicum*
panDfwd	tatacatatgcatttaaatatgttaaaatctaaa	*Nde*I/*C. acetobutylicum*
panBrev	tatagctagcttatttggtaatttcgtctataatg	*Nhe*I/*C. acetobutylicum*
panCrev	tatagctagcttatatattccaagtgaaattatctataagg	*Nhe*I/*C. acetobutylicum*
panDrev	Tatagctagcttagccaattgtcc	*Nhe*I/*C. acetobutylicum*
panB2fwd	Tataactagtatgaaaaataccactgaaac	*Spe*I/*C. acetobutylicum*
Biotin		
bioYDAF1	tatacatatgattatggtttctatttttgc	*Nde*I/*C. acetobutylicum*
bioYDAR1	tatagctagcttattcaagatactgctctatactttc	*Nhe*I/*C. acetobutylicum*
bioBDFF1	tatacatatgagcaatattttgaaacatatcg	*Nde*I/*D. carboxidivorans*
bioBDFR1	tatagctagcttaaatcaatcccagttc	*Nhe*I/*D. carboxidivorans*
bioHCAF1	tatacatatgttggaaaaacagagattaatcc	*Nde*I/*D. carboxidivorans*
bioHCAR1	tatagctagcttaatctattccaaaatagttattaattgc	*Nhe*I/*D. carboxidivorans*
Thiamine		
thiCpurFfwd	gcagggcccaatacgattatctcctttc	*Apa*I/*C. ragsdalei*
thiCpurFrev	gcatcctgcaggtaaattttgttcttcatt	*Sbf*I/*C. ragsdalei*
ACE		
SHAF	tccgcggaagggcattttctactttcttc	*Sac*II/*C. ljungdahlii*
SHARd	agcggccgccacatcttcagaaattataattttttctcc	*Not*I/*C. ljungdahlii*
SHARc	agcggccgcttatttgaatactctacttccaggc	*Not*I/*C. ljungdahlii*
GHAF	tatgctagcgtcgaaaaaatcaatgcacgatgc	*Nhe*I/*C. ljungdahlii*
GHAR	aggcgcgcctactgggtaacggaacctg	*Asc*I/*C. ljungdahlii*
CH21thlF1	tatagctagcccgctttttaacaaaatatattg	*Nhe*I/pMTL8xxx2
CH21thlR1	tataactagtggggtaacagataaaccatttc	*Spe*I/pMTL8xxx2
cacpyreF	atggaacaatacaaacaagaatttatag	*/ C. acetobutylicum*
cacpyreR	gagtactataaagagtatggagtaaaatag	*/ C. acetobutylicum*
HiFi assembly		
P1F	atgaaatagattgaaatgagcaatattttgaaacatatc	/ pMTL-FJAL22
P2R	gctactacacttaaatcaatcccagttcttttc	/ pMTL-FJAL22
P3F	ggattgatttaagtgtagtagcctgtgaaataag	/ pMTL-FJAL23
P4R	ttgattttttcgacgctagcttaatctattccaaaatagttattaattgc	/ pMTL-FJAL23

Genomic DNA of *Desulfotomaculum nigrificans* CO-1-SRB (Parshina et al. 2005; Visser et al. 2014) was obtained from DSMZ (DSM14880). The DNeasy blood and tissue kit (Qiagen) were used to isolate genomic DNA from *C. acetobutylicum*. Genomic DNA of *C. ragsdalei* was extracted as follows. A 100-mL sample of an exponentially growing culture was harvested (4000×*g*, 15 min, 4 °C), washed with potassium phosphate buffer (10 mM, pH 7.5) and re-suspended in 1.9-mL STE buffer (50 mM Tris-HCl, pH 8.0, 1 mM EDTA, 200 mM sucrose). The cells were incubated for 30 min with 300 μL of lysozyme (100,000 U) at 37 °C. The lysis step was followed by a 10-min incubation with 10% (*w*/*v*) SDS. The RNA was digested at room temperature by adding 240 μL of 0.5 M EDTA (pH 8.0), 20 μL of 1 M Tris-HCl (pH 7.5) and 100 μL of RNase A. Then, 100 μL proteinase K (0.5 U) were added and proteolysis took place for 3 h at 37 °C. Finally, 600 μL of sodium perchlorate (5 M) was added, followed by a phenol-chloroform extraction and an isopropanol precipitation.

Sanger sequencing was used to validate DNA sequences using plasmid or PCR products as template (by Eurofins Genomics or Source Bioscience). Plasmids used in this study are listed in Table 3.

**Table 3:** Plasmids constructed in this study

Plasmid	Backbone ^a^	Functional module	F-primer	R-primer
ACE				
pANNE98	pMTL84141	dSHA	SHAF	SHARd
pANNE99	pMTL84141	GHA	GHAF	GHAR
pMTL-AMH100	pANNE99	ACE PyrE deletion		
pMTL-AMH101	pMTL-CH14	ACE PyrE deletion		
pMTL-AMH102	pMTL84141	ACE PyrE wt-restore	SHAF	GHAR
pMTL-AMH103	pANNE99	ACE PyrE correction	SHAF	SHARc
pMTL-CH14	pMTL84151	*C. acetobutylicum pyrE* ^b^	cacpyreF	cacpyreR
pMTL-CH20	pMTL84151	ACE PyrE correction		
pMTL-CH21	pMTL-CH20	ACE PyrE correction + BB2		
pMTL-CH23	pMTL-CH21	ACE PyrE correction + P_thl_ + BB2	CH21thlF1	CH21thlR1
Pantothenate				
pMTL-FJAL3	pMTL82152	P_thl_-panBCD	panBfwd	panDrev
pMTL-FJAL4	pMTL83153	P_fdx_-panCD	panCfwd	panDrev
pMTL-FJAL5	pMTL83153	P_fdx_-panBC	panBfwd	panCrev
pMTL-FJAL6	pMTL82252	P_thl_-panB	panBfwd	panBrev
pMTL-FJAL7	pMTL82252	P_thl_-panC	panCfwd	panCrev
pMTL-FJAL8	pMTL82252	P_thl_-panD	panDfwd	panDrev
pMTL-FJAL10	pMTL-CH23	P_thl_-panBCD	panB2fwd	panDrev
Biotin				
pMTL-FJAL20	pMTL82252	P_thl_-bioYDA	bioYDAF1	bioYDAR1
pMTL-FJAL21	pMTL82252	P_thl_-bioBDF	bioBDFF1	bioBDFR1
pMTL-FJAL22	pMTL83153	P_fdx_-bioHCA	bioHCAF1	bioHCAR1
pMTL-FJAL23	pMTL-CH23	P_thl_-bioBDF-P_fdx_-bioHCA	HiFi assembly	
pMTL-FJAL24	pMTL83251	P_thl_-bioBDF-P_fdx_-bioHCA		
Thiamine				
pMTL-BAS1	pMTL85242	P_native_-thiC-purF	thiCpurFfwd	thiCpurFrev

^a^pMTL8xxxx vectors described by Heap et al. 2009

^b^Contains *C. acetobutylicum pyrE* gene in backbone downstream of *catP*

### Plasmid DNA transfer into *Clostridium* spp.

Plasmids were introduced into *C. autoethanogenum* by conjugation from the *E. coli* donor strain CA434 as previously described (Liew et al. 2017). In contrast, plasmids were introduced into *C. ljungdahlii* by electroporation as follows. Electrocompetent cells of *C. ljungdahlii* were prepared from a culture grown in 300-mL YTF medium in exponential growth phase with an OD600 of 0.2–0.3. Cells were pelleted and washed twice in 30-mL anaerobic SMP5.8 buffer (1 mM sodium phosphate pH 5.8, 1 mM MgCl_2_, 270 mM sucrose) in anaerobic 50-mL conical polypropylene tubes using an 5430R centrifuge (Eppendorf, Germany) placed inside a model B vinyl anaerobic chamber (Coy Laboratory Products, MI, USA) at 4552×*g* for 5′ at ambient temperature. Cell pellets were resuspended by vortexing and then pooled. After the second wash, the cell pellet was resuspended in 1.8-mL SMP5.8 buffer and 0.2 mL DMSO, divided over 2-mL polypropylene screw cap microtubes (Sarstedt) in 0.11-mL aliquots, flash frozen in liquid nitrogen and stored at − 80 °C. For transformation of electrocompetent cells 100-μL suspension was transferred to a 2-mm electroporation cuvette (Biorad) with 10-μL plasmid solution and pulsed (1000 V, 50 μF, 200 Ohm) with a GenePulser Xcell electroporation instrument equipped with PC and CE modules (Biorad). The pulsed cell suspension was transferred to 5-mL anaerobic YTF recovery medium, incubated for 20 h and pelleted. Cell pellets were resuspended in 100-μL spent recovery medium and plated on solid YTF medium with appropriate selection marker. Colonies would form over 2–3 days. RCLJU medium was used instead of YTF medium and an EBA21 centrifuge (Hettich, Germany) placed inside a Don Whitley MG1000 anaerobic cabinet for the partial deletion of *pyrE* in *C. ljungdahlii* described below in the procedure detailed above. RCLJU medium was composed as MBM medium with YE, except that acid and alkaline trace element solutions (Stams et al. 1993) were used instead.

### Construction of expression vectors

Genes that were identified as necessary to complete biotin or pantothenate metabolic pathways were amplified by PCR with Q5 DNA polymerase, using genomic DNA of donor species as a template. Similarly, genes for completion of thiamine pathway were amplified using iProof HF DNA polymerase (Bio-Rad) and FailSafe 2× PCR premix E buffer (Epicentre). PCR products were digested with endonucleases indicated for each primer pair (Table 2), purified by agarose gel electrophoresis, extracted from gel, and subsequently ligated to a pMTL80000 series shuttle vector (Heap et al. 2009) that was digested with the same endonuclease pair and extracted from gel. Ligation products were used to transform *E. coli* TOP10 (Invitrogen), and transformants with plasmid were selected on solid LB media with appropriate antibiotic selection marker. Plasmid DNA was isolated and submitted for Sanger sequencing to validate the nucleotide sequence of the cloned genes. Only expression vector pMTL-FJAL24 was obtained by subcloning a DNA fragment from another plasmid (pMTL-FJAL23, described below) into pMTL83251 using *Not*I and *Nhe*I.

### Creation of ACE vectors

**Fig. 1:**
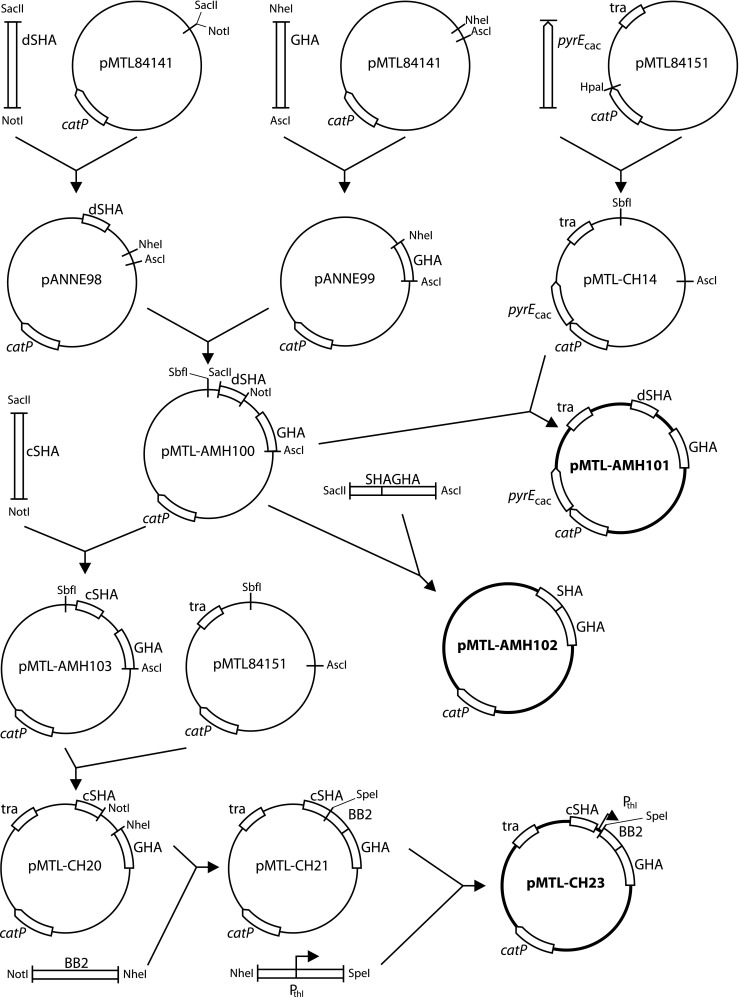
Schematic representation of subcloning of the ACE vectors pMTL-AMH101 for deletion of pyrE, pMTL-CH23 for correction of pyrE and pMTL-AMH102 for resaturation of the pyrE locus. Full detail is provided in “Material and methods”. Linear fragments represent PCR products, except for the BB2 fragment which was synthesised. Subcloning steps used the indicated restriction endonucleases. Sizes of depicted molecules do not relate to molecular size

Genes were integrated in the chromosomes in strains of *C. autoethanogenum* and *C. ljungdahlii* using ACE in combination with the *pyrE* allele (Heap et al. 2012). A set of ACE vectors for *pyrE* deletion, restoration and correction was constructed as depicted (Fig. 1) as follows. The long region of homology (GHA) and the short region of homology needed to create the partial *pyrE* deletion (dSHA) were amplified by PCR with indicated primers (Table 3) and each product ligated into a pMTL84141 vector after digestion with the indicated restriction endonucleases (Table 2) and treatment of the vector with antarctic alkaline phosphatase (AAP). Ligation products were used to transform *E. coli* XL1-blue chemical competent cells (Agilent). Successful transformants carried vector pANNE98 with dSHA or pANNE99 with GHA (Table 3). Both homology regions were combined in one vector after restriction digestion of both vectors with *Nhe*I and *Asc*I, extraction of restriction fragments from gel and ligation of the GHA region into pANNE98 backbone. Ligation products were used to transform *E. coli* XL1-blue. Successful transformants carried vector pMTL-AMH100. The vector pMTL-AMH101 was created after it was found that with pMTL-AMH100, plasmid loss was not obtained in the process required to create the partial *pyrE* gene deletion (data not shown). Vector pMTL-AMH101 contained *pyrE* of *C. acetobutylicum* directly downstream of *catP* on the vector backbone to enforce plasmid loss during 5-FOA selection. To create pMTL-AMH101, the functional module of pMTL-AMH100 was subcloned into pMTL-CH14 using *Sbf*I and *Asc*I. The plasmid pMTL-CH14 itself was obtained after ligating the PCR-amplified *pyrE* gene of *C. acetobutylicum* with indicated primers (Table 3) in pMTL84151 after digestion with HpaI, transformation of *E. coli* TOP10. In successful pMTL-CH14 transformants, the inserted *pyrE* was of the same orientation as catP.

The vectors pMTL-AMH102, with native regions of short and long homology adjoined (SHAGHA) to restore the partial chromosomal *pyrE* deletion to wildtype, and pMTL-AMH103, with the short region of homology to correct the partial chromosomal *pyrE* deletion (cSHA) and integrate a genetic payload at the same time, were also derived from pMTL-AMH100, as follows. The regions of homology were amplified with indicated primers (Table 3) by PCR, digested with indicated restriction endonucleases (Table 2) and extracted from gel. The vector pMTL-AMH100 was digested with the same pairs of restriction endonucleases and extracted from gel, before ligation of the processed PCR fragments, and transformation of *E. coli* XL1-blue with ligation products. The functional module of pMTL-AMH103 was then subcloned using *Sbf*I and *Asc*I into pMTL84151 to obtain the vector pMTL-CH20 which was suitable for transconjugation. A promoter to drive expression of a gene payload of the integration vector was introduced in two successive steps. First, a synthetic BioBrick2 (Knight 2003) fragment (topology: 5′-BB2prefix[*Eco*RI-*Not*I-*Spe*I]-synthethic_gene-BB2suffix[*Nhe*I-*Not*I-*Pst*I]) was subcloned in pMTL-CH20 using *Not*I and *Nhe*I, resulting in pMTL-CH21. Then, a PCR product, with P_thl_ amplified using indicated primers (Table 3) from a suitable pMTL80000 vector that carried this promoter as template, was digested with *Spe*I (restriction site originating from the BB2 prefix) and *Nhe*I (compatible to *Spe*I) and ligated in plasmid pMTL-CH21 that was only digested with *Spe*I and treated with AAP. Successful *E. coli* Top10 transformants contained pMTL-CH23.

### Creation of partial *pyrE* deletion strains

The vector pMTL-AMH101 was used to delete the C-terminus of *pyrE* in *C. autoethanogenum* and *C. ljungdahlii* as follows. After transformation of *C. autoethanogenum* with pMTL-AMH101, thiamphenicol-resistant clones were restreaked onto solid YTF + FU (FOA and uracil) medium. Colonies that formed were then restreaked on solid MBM medium (without uracil), MBM + U medium (with uracil) and MBM + UTm medium (with uracil and thiamphenicol). Clones that grew on MBM + U medium but not on MBM and MBM + UTm medium were selected for further work. Deletion of the 3′ end of *pyrE* in the chromosome of obtained clones was validated by isolation of genomic DNA, amplification of *pyrE* locus by PCR and Sanger sequencing of the PCR product.

After transformation of *C. ljungdahlii*, thiamphenicol-resistant transformants were used to inoculate liquid rCLJU medium (composed as RCLJU except with 1 g/L YE) with thiamphenicol. From the resultant cultures, 100 μL was spread on solid CCLJU + FU medium (composed as RCLJU, except with CAS instead of YE) and colonies that formed were restreaked on solid CCLJU + FU medium. Colonies that then formed were restreaked on solid CCLJU medium (without uracil), solid RCLJU medium and solid RCLJU + Tm medium. Deletion of *pyrE* was validated for clones that only grew on RCLJU medium (without Tm).

### Chromosomal integration of heterologous genes

**Fig. 2:**
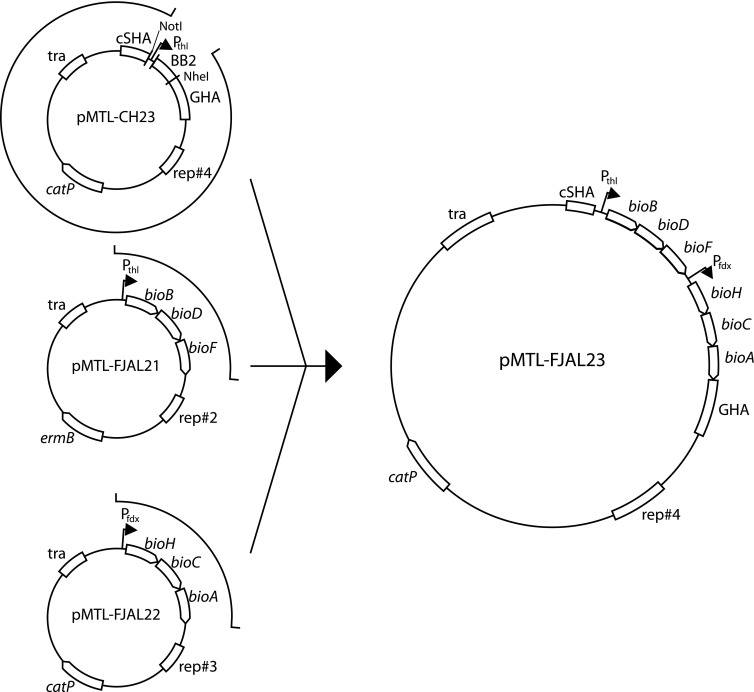
Schematic representation of HiFi assembly of pMTL-FJAL23 integrative ACE vector. Full detail is provided in the text. The indicated sections of depicted plasmids were assembled. For pMTL-FJAL21 and pMTL-FJAL22, the sections were amplified by PCR with overlapping primers. The section forming the backbone of pMTL-CH23 was obtained after digestion of the plasmid with *Not*I and *Nhe*I. Sizes of depicted molecules do not relate to molecular size

Genes were integrated in the chromosomes of *C. autoethanogenum* Δ*pyrE* and *C. ljungdahlii* Δ*pyrE* by ACE. A vector (pMTL-FJAL10) for integration of *panBCD* was constructed similar to the construction of expression vectors. PCR products were subcloned into pMTL-CH23 using *Spe*I and *Nhe*I. A vector for integration of *bioBDF-bioHCA* (pMTL-FJAL23) was constructed by HiFi DNA assembly from three DNA fragments (F1, F2 and F3) using four primers (P1F, P2R, P3F and P4R) following manufacturer’s instructions (Fig. 2). Fragment F1 was amplified by PCR from pMTL-FJAL21 as template with primers P1F and P2R (Table 2). Fragment F2 was amplified by PCR from pMTL-FJAL22 as template with primers P3F and P4R (Table 2). Plasmid pMTL-CH23 digested with *Not*I and *Nhe*I and extracted from agarose gel served as fragment F3. Validated integration vectors (pMTL-FJAL10 and pMTL-FJAL23) were used to transform Δ*pyrE* strains and transformants or transconjugants selected on medium with antibiotic and uracil supplemented, then restreaked on medium without uracil and medium with antibiotic in parallel, until antibiotic resistance was lost, to select for chromosomal integrants that lost the integrative plasmid. The DNA base sequence of the integrated heterologous genes was validated.

## Results

### Phenotypic analysis

Growth experiments in various defined media that lacked specific amino acids or vitamins were performed to establish for which compounds *C. ljungdahlii* and *C. autoethanogenum* were auxotrophic. A MES-buffered mineral base medium with vitamins (MBM) to which amino acids, YE or CAS were added, or from which vitamins were omitted, was used. Cysteine or sodium sulfide served as reducing agent and source of sulfur. Both strains grew in media lacking YE, CAS or any other source of amino acids (including cysteine) when B-vitamins were supplemented (Table 1). Auxotrophy for vitamins was initially tested in MBM + CAS from which one vitamin was omitted, for each vitamin. Growth was not observed in serial dilutions in medium lacking pantothenate, biotin or thiamine. In further experiments in MBM medium with sodium sulfide, growth was only obtained if pantothenate, biotin and thiamine were present (Table 1). The outcome of growth experiments with wild type, and also the derived strains mentioned below, was the same if fructose or CO was used as growth substrate and no obvious differences in cell morphology was observed by phase contrast microscopy. Also, the final optical densities of cultures that grew were very similar (data not shown), either due to another compound in the medium than CO or fructose being growth limiting, or due to the limitations of the used spectrophotometric technique that did not allow dilution of the samples.

### Metabolic pathway analysis

**Fig. 3:**
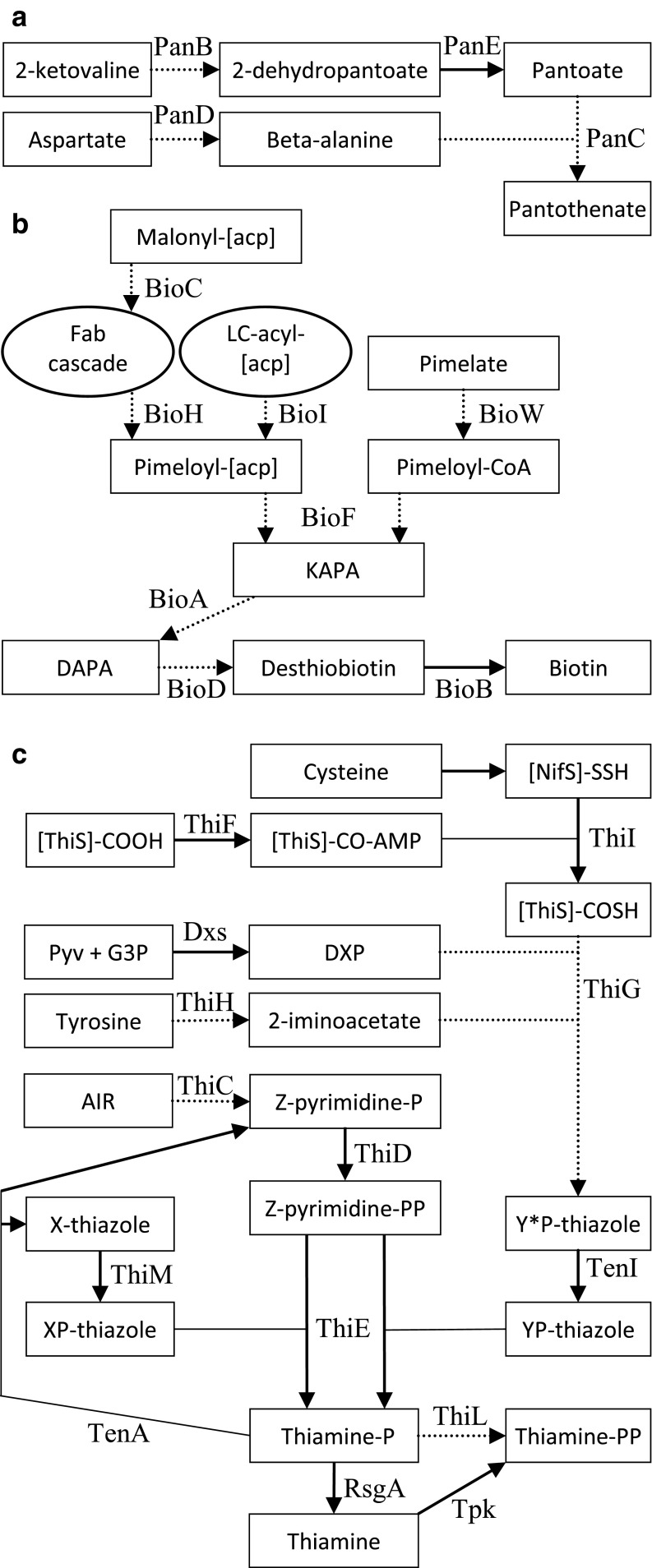
The incomplete pantothenate, biotin and thiamine biosynthesis pathways of *C. autoethanogenum* and *C. ljungdahlii*. Missing steps are indicated by a dotted line. Details on enzymes are provided in the text. **a** Pantothenate biosynthesis pathway. **b** Biotin biosynthesis pathway. **c** Thiamine biosynthesis pathway. Abbreviations: [acp] = acyl-carrier protein; Fab = fatty acid biosynthesis; LC = long chain; KAPA = 8-amino-7-oxononanoate; DAPA = 7,8-diaminononanoate; Pyv = pyruvate; G3P = glyceraldehyde-3-phosphate; DXP = 1-deoxy-D-xylulose 5-phosphate; AIR = aminoimidazole ribotide; X = 4-methyl-5-[(2-hydroxy)-ethyl]-2,5-dihydro-; XP = 4-methyl-5-[(2-phosphonooxy)-ethyl]-2,5-dihydro-; Y*P* = 2-carboxy-4-methyl-5-[(2-phosphonooxy)-ethyl]-2,5-dihydro-; Y*P = 2-carboxy-4-methyl-5-[(2-phosphonooxy)-ethylidene]-2,5-dihydro-; Z = 4-amino-2-methyl-5-(diphosphooxymethyl)-

Bioinformatic analysis was used to investigate whether incomplete metabolic pathways could account for auxotrophy in *C. autoethanogenum* and *C. ljungdahlii*. It was found that the whole genome metabolic models for both strains, iCLAU786 and iHN637, respectively, contained functional pathways to pantothenate and iCLAU786 also to thiamine. This was in disagreement with our growth experiments. Reconstruction of metabolic pathways for pantothenate, biotin and thiamine was therefore performed by manual curation of whole genome sequences and revealed that one or more genes were missing for each biosynthetic pathway and that these deficiencies were the same in both microorganisms. These pathways are discussed in more detail in “Discussion”; here, we mention only the affected steps. The pantothenate biosynthesis pathway lacked orthologs for *panB*, *panC* and *panD* (Fig. 3a). Of the biotin biosynthesis pathway, only *bioB* was present and no orthologs for *bioW*, *bioI*, *bioD*, *bioF*, *bioH*, *bioC* and *bioH* were found (Fig. 3b). The thiamine biosynthesis pathway lacked orthologs for *thiG*, *thiH* and *thiC* (Fig. 3c). In addition, no clear ortholog for TenI was present in the thiamine biosynthesis pathway. Two putative orthologs for *B. subtilis* TenI, annotated as *thiE1* and *thiE2*, contained relative poor query coverage (49% and 67%) and sequence identity (48% and 31%) as obtained with BLAST. While genes annotated as *thiS* and *thiF* were present, however only part of the genes showed homology with known *thiS* and *thiF* genes. Gene context of *thiS* and *thiF* genes did not directly hint at thiamine biosynthesis. Introduction of the steps that were missing in the vitamin biosynthesis pathways of the metabolic models iCLAU786 and iHN637 resulted in the production of pantothenate, thiamine and biotin by these models (Online Resource 1).

### Genetic engineering of pantothenate prototrophy

*C. acetobutylicum*, as a phylogenetically closely related microorganism, was used as source for the genes needed to introduce pantothenate prototrophy in *C. autoethanogenum* and *C. ljungdahlii*. Modular pMTL80000 vectors with P_fdx_ or P_thl_ promoter (Heap et al. 2009) and downstream *panB*, *panC* and/or *panD* genes of *C. acetobutylicum* were created (Table 3) and introduced into *C. autoethanogenum* and *C. ljungdahlii* to test whether various combinations of these genes could confer pantothenate prototrophy on the target microorganisms. Plasmid-bearing strains were inoculated in medium lacking pantothenate to test their viability. Strains that harboured a plasmid that carried all three genes were able to grow in medium lacking pantothenate (Table 4). This plasmid was maintained for at least eight serial transfers in medium without antibiotic. Also, strains that carried two plasmids that together specified all three genes were able to grow in medium without pantothenate and were maintained for at least eight serial transfers in absence of antibiotic (Table 4). It was also of interest to see whether pantolactone and beta-alanine could serve as provitamins and replace the need for *panB* or *panD*, respectively (Fig. 1a). Growth was detected for plasmid-bearing strains that possessed *panC* in medium that contained pantolactone and beta-alanine, and in those strains that carried *panB* and *panC* in medium supplemented with pantolactone, as well as in cells carrying *panC* and *panD* in medium containing beta-alanine (Table 4).

**Table 4:** Growth of recombinant strains in defined media

Genotype	Recombinant molecule	Medium				
		Supplement	MBM	MBM-P	MBM-B	MBM-T
Wt			+	–	–	–
*panBCD*	pMTL-FJAL3		+	+		
*panBCD*	C		+	+		
*panB*	pMTL-FJAL6		+	–		
*panC*	pMTL-FJAL7		+	–		
*panD*	pMTL-FJAL8		+	–		
*panBC*	pMTL-FJAL5		+	–		
*panCD*	pMTL-FJAL4		+	–		
*panBC* + *panD*	pMTL-FJAL5 + pMTL-FJAL8		+	+		
*panCD* + *panB*	pMTL-FJAL4 + pMTL-FJAL6		+	+		
*panBC*	pMTL-FJAL5	b-alanine	+	+		
*panCD*	pMTL-FJAL4	pantolactone	+	+		
*panC*	pMTL-FJAL7	b-alanine + pantolactone	+	+		
*bioYDA*	pMTL-FJAL20		+		–	
*bioYDA*	pMTL-FJAL20	Pimelate	+		+	
*bioBDF*	pMTL-FJAL21		+		–	
*bioHCA*	pMTL-FJAL22		+		–	
*bioBDF* + *bioHCA*	pMTL-FJAL21 + pMTLFJAL22		+		+	
*bioBDF_bioHCA*	pMTL-FJAL24		+		+	
*bioBDF_bioHCA*	C		+		+	
*panBCD* + *bioBDF_bioHCA*	pMTL-FJAL3+ pMTL-FJAL24		+	+	+	
*thiC-purF*	pMTL-BAS1		+			+

*c* chromosome, *P* except pantothenate, *B* except biotin, *T* except thiamine

### Chromosomal integration of *panBCD*

A partial *pyrE* deletion mutant of *C. autoethanogenum* and *C. ljungdahlii* was created and then used to integrate *panBCD* genes into the chromosome using ACE, concomitant with restoration of uracil prototrophy. Integrants of both species designated as strains PANTS were able to grow without pantothenate supplementation of the medium (Table 1, Table 4).

### Genetic engineering of biotin prototrophy

Initially, *C. acetobutylicum* was used as a potential source of the genes needed to confer biotin prototrophy on *C. autoethanogenum* and *C. ljungdahlii*. *C. acetobutylicum* has been reported to grow in medium without biotin supplemented (Li et al. 2013), but only contains genes for BioB, BioA, BioD and BioY (Yang et al. 2016).

However, overexpression of *bioYDA* significantly improved its growth (Yang et al. 2016). Accordingly, an expression vector was created that carried the *bioYDA* genes of *C. acetobutylicum* (CA_c1360-c1362) and introduced into *C. autoethanogenum* by conjugative plasmid transfer. However, wild-type and transconjugant strains of *C. autoethanogenum* were not able to grow in medium without biotin. If pimelate was added to the medium as a provitamin, then the transconjugant strains were able to grow, but not the wild-type strains (Table 4).

The strict anaerobic, Gram-positive, carboxydotrophic bacterium *Desulfotomaculum nigrificans* strain CO-1-SRB was used as source for genes to introduce a complete biosynthesis pathway for biotin into *C. autoethanogenum*. The needed *bio* genes for biotin biosynthesis in this strain were conveniently located in a single operon *bioBDFHCA* (Desca_0053–0058). Two expression vectors were created that carried *bioBDF* downstream of P_thl_ (pMTL-FJAL21) and *bioHCA* downstream of P_fdx_ (pMTL-FJAL22). Both vectors were introduced into *C. autoethanogenum* by conjugative plasmid transfer, involving the use of two *E. coli* donors carrying one or other of the plasmids. It has to be noted that the success rate for this simultaneous conjugation was low and only resulted in a few colonies in the third attempt. Transconjugants were able to grow in medium without biotin (Table 4).

For chromosomal integration of all the biotin biosynthesis genes, a single integration vector was created using NEB HiFi assembly and plasmids pMTL-FJAL21, pMTL-FJAL22 and pMTL-CH23 as templates. A single plasmid avoids the need for multiple iterations of ACE, although its larger size is likely to result in lower rates of plasmid transfer. Nonetheless, transconjugants were successfully isolated for *C. autoethanogenum* in which integration had taken place and this strain was designated strain BIO. Moreover, these integrants were able to grow on medium without biotin supplementation (Table 4).

### Combined pantothenate and biotin prototrophy in *C. autoethanogenum*

An expression vector pMTL-FJAL24 that carried the P_thl_-*bioBDF*-P_fdx_-*bioHCA* module was created and transferred into *C. autoethanogenum* by conjugation simultaneously with pMTL-FJAL3 that carried P_thl_-*panBCD*. Cells carrying these two plasmids were able to grow in medium without biotin and pantothenate supplementation, although the observed growth rate was reduced. This strain was designated *C. autoethanogenum* biopants (Table 1, Table 4).

### Genetic engineering of thiamine prototrophy

Comparative genomics revealed that in contrast to *C. autoethanogenum* and *C. ljungdahlii*, a *thiC* gene is present in the closely related acetogenic species *C. ragsdalei*, with directly downstream a *purF* gene (Bengelsdorf et al. 2016). PurF is a phosphoribosyl pyrophosphate (PRPP) amidotransferase that synthesises phosphoribosylamine (PRA), the first step in purine biosynthesis and a precursor of aminoimidazole ribotide (AIR), the substrate of ThiC. Despite the presence of *purF* in the genomes of *C. autoethanogenum* and *C. ljungdahlii*, and potentiality of alternative paths to AIR (Bazurto et al. 2016), *purF* was included in the plasmid constructed to complement thiamine biosynthesis. A vector with the *thiC-purF* genes and the directly upstream intergenic region, to include the native promoter, was constructed (pBAS001/pMTL8524-thiC-purF) and introduced into *C. autoethanogenum* and *C. ljungdahlii*. Transformants were able to grow in medium without thiamine in successive transfers, while growth of the wild-type and plasmid control strain ceased after two subculture steps.

## Discussion

Growth experiments indicated that C. *autoethanogenum* and *C. ljungdahlii* are auxotrophic for pantothenate, biotin and thiamine. Also, genomic analysis revealed that both microorganisms lacked the same genes in their biosynthetic pathways. Complementation of pantothenate (in *C. ljungdahlii* and *C. autoethanogenum*), biotin (in *C. autoethanogenum*) and thiamine (in *C. ljungdahlii* and *C. autoethanogenum*) biosynthetic pathways is discussed.

### Pantothenate biosynthesis

Pantothenate is a key precursor for the synthesis of coenzyme A (CoA) and produced from aspartate and 2-ketovaline, an intermediate of the valine biosynthesis pathway, in four enzymatic steps catalysed by PanB, C, D and E (Fig. 3a) (Leonardi and Jackowski 2007). The pantothenate biosynthesis pathway of *C. autoethanogenum* and *C. ljungdahlii* lacked genes for ketopantoate hydroxymethyltransferase (PanB), pantothenate synthetase (PanC), and aspartate carboxylase (PanD). The *panBCD* gene cluster of *C. acetobutylicum* (CA_C2914-2916) was successfully used to complement the pantothenate biosynthesis pathway of *C. ljungdahlii* and *C. autoethanogenum*, from plasmid and when integrated in the genome. Transformations with single genes and pairs of genes demonstrated that pantolactone could substitute for *panB* and beta-alanine for *panD*. If pantolactone and beta-alanine were supplied simultaneously, only *panC* was needed in addition for growth in the absence of pantothenate from the medium. Since yeast extract does not contain sufficient pantothenate to support growth of *C. autoethanogenum* and *C. ljungdahlii*, the pantothenate biosynthesis genes can be used as selectable marker in media that lack pantothenate, but contain yeast extract to promote rate of colony formation. Additionally, the three genes can be used independently in combination with beta-alanine and/or pantolactone to allow for cloning strategies that require sequential transfers of multiple vectors.

### Biotin biosynthesis

Of the biotin biosynthetic pathway, only *bioB* and *bioY* were present (Fig. 3b). BioB catalyses the conversion of desthiobiotin to biotin, the final step in biotin biosynthesis. BioY is a biotin transporter. Genes for conversion of the pimeloyl moiety to biotin (*bioD*, *bioA* and *bioF*), which are conserved across all domains of life, were not found. Also, dedicated genes for the synthesis of the pimeloyl moiety (*bioW* and *bioI* or *bioH* and *bioC*) were absent (Fig. 1b). The steps for the conversion of the pimeloyl-moiety BioC and BioH are key enzymes in the biotin synthesis pathway of *E. coli*, which “hijacks” the fatty acid biosynthesis cascade to elongate the carbon chain of acyl-carrier protein ([acp]) bound malonyl methyl ester that is formed by BioC (Lin et al. 2011). BioH salvages pimeloyl-[acp] methyl ester from the cascade and converts it to pimeloyl-[acp]. BioI and BioW are used by *B. subtilis* for biosynthesis of the pimeloyl moiety of biotin (Manandhar and Cronan 2017). BioI utilises molecular oxygen to cleave pimeloyl-acp from long chain acyl-[acp] molecules and is as such unsuitable for use in an anaerobe. While *bioI* was shown not essential in *B. subtilis*, its deletion resulted in a biotin bradytroph. BioW was reported essential in *B. subtilis* and converts pimelate to pimeloyl-CoA (Manandhar and Cronan 2017). Furthermore, it was found that BioF of *B. subtilis* specifically used pimeloyl-CoA, whereas *E. coli* BioF also used pimeloyl-[acp] (Manandhar and Cronan 2018). Recently, it was reported that *C. acetobutylicum* was able to grow in defined medium without biotin (Li et al. 2013; Yang et al. 2016), although carry-over of biotin in the method used cannot be excluded. Only *bioB*, *bioY*, *bioD* and *bioA* were identified in its genome and not *bioW*, *bioI* or *bioF* (Yang et al. 2016). We were also unable to identify *bioC* and *bioH* (data not shown). Accordingly, the *bioYDA* gene cluster of *C. acetobutylicum* was introduced into *C. autoethanogenum*. The transconjugants obtained were not, however, prototrophic for biotin. Transconjugants carrying the *bioYDA* genes did grow when pimelic acid was added to medium lacking biotin, while wild-type *C. autoethanogenum* did not grow. Apparently, the gene cluster *bioYDA* was sufficient for *C. autoethanogenum* to convert pimelic acid to biotin, despite the absence of known genes for BioW and BioF. It remains to be investigated whether *C. autoethanogenum* contains alternative genes to catalyse these steps or that 7-keto-8-aminopelargonic acid (KAPA), the substrate of BioA, is formed from pimelic acid through a completely different mechanism (Fig. 1b). Full biotin prototrophy of *C. autoethanogenum* was obtained after transformation with gene clusters *bioBDF* and *bioHCA* of the Gram-positive, strictly anaerobic, carboxydotrophic *D. nigrificans* CO-1-SRB. These genes also conveyed biotin prototrophy when integrated in the genome (Table 4).

Prototrophy for pantothenate and biotin was combined successfully by transfer of plasmids carrying *panBCD* and *bioBDF-bioHCA* gene clusters to *C. autoethanogenum*. Growth of transconjugants was noticeably slower (data not shown) and likely due to the need for the cells to maintain two plasmids of which one of considerable size (~ 13 kbp). Transfer of both plasmids was unsuccessful in *C. ljungdahlii* and most likely due to the low efficiency of the used transformation method. All generated prototroph strains were also able to grow with CO as sole carbon and energy source (instead of fructose).

### Thiamine biosynthesis

Thiamine biosynthesis follows a complex pathway in which a pyrimidine and a thiazole moiety are joined to form thiamine pyrophosphate (TPP), the active form of thiamine, and requires at least 10 enzymes in anaerobic bacteria (Fig. 3). The thiazole is formed through activity of ThiFSGH, ThiI and Dxs and the pyrimidine through ThiC and ThiD. The pyrimidine and thiazole are coupled by ThiE to form thiamine monophosphate (TMP) and in *E. coli* is phosphorylated to TPP by ThiL (Begley1999). In some microorganisms, ThiL is lacking and instead TMP is first dephosphorylated by RgsA to thiamine, that in turn is converted by thiamine pyrophosphokinase (Tpk) to TPP. Additional enzymes associated with the thiamine biosynthesis pathway exist that play a role in thiamine salvage pathway (TenA and ThiM) or that convert (by TenI) the thiazole (Y*P-thiazole) produced by thiazole synthase (ThiG) to a tautomer (YP-thiazole) that is more readily used by thiamine phosphate synthase (ThiE) (Hazra et al. 2009, 2011). The thiamine biosynthesis pathway of *C. autoethanogenum* and *C. ljungdahlii* was found to lack genes for the key enzymes 2-iminoacetate synthase (ThiH), ThiG and phosphomethylpyrimidine synthase (ThiC) (Fig. 3c). Two paralogs for ThiE were found, ThiE1 and ThiE2, and it is likely one of these functions as TenI (Rodionov et al. 2002). The lack of *thiC*, *thiG* and *thiH* is common among enteric *Clostridia* and instead an operon with thiamine salvage pathway genes and transporters for the pyrimidine and thiazole moieties may be present (Rodionov et al. 2002), as was the case for *C. autoethanogenum* and *C. ljungdahlii* that contained a ThiDME operon, a transporter for the thiamine (ThiW) and pyrimidine (ThiYXZ) moiety, and must contain an uptake system for thiamine as the wild-type strains are able to grow in minimal medium with thiamine supplemented.

However, we found that the thiamine biosynthesis pathway could be completed with just the *thiC*-*purF* operon from *C. ragsdalei* in minimal medium that lacked thiamine and precursors of the pyrimidine and thiazole moieties. This indicates that either unknown genes or an unknown pathway exists that replaces ThiG and ThiH function.

*C. autoethanogenum* and *C. ljungdahlii* were found to be auxotrophic for pantothenate, biotin and thiamine. Suitable genes to complement biosynthesis pathways were identified and successfully used to confer prototrophy for each of these three vitamins in vivo. This is the first report of chromosomal integration of genes in the chromosomes of *C. autoethanogenum* and *C. ljungdahlii* by ACE (Heap et al. 2012). Study of the biosynthetic pathways for these vitamins revealed that the available whole genome metabolic models iHN637 and iCLAU786 (Nagarajan et al. 2013; Marcellin et al. 2016) erroneously already contained pathways for pantothenate and thiamine production. The models used automatically annotated genome sequences that were not manually curated. Interestingly, iCLAU786 produced pantothenate with just panBC while we showed in vivo that panD was essential as well. The beta-alanine that is produced by panD was in the model derived from uracil (data not shown). Both models were amended with complete pathways to reflect the prototrophy for pantothenate, biotin and thiamine (Online Resource 1), however with the thiamine biosynthesis pathway as it appears in iCLAU786 and that includes a ThiGH thiazole synthase reaction. In reality, it remains unclear how the thiazole is formed as we found genes for ThiG and ThiH to be lacking from the genome sequences and only introduction of thiC and purF resulted in complementation of thiamine biosynthesis. Similarly, biotin could be synthesised from pimelate without the apparent need of bioW and bioF. Likely, these metabolic steps are carried by still unidentified genes that are present in these strict anaerobic Gram-positive acetogens.

## Electronic supplementary material

ESM 1 (TXT 620 bytes)

ESM 2 (SBML 820 kb)

ESM 3 (SBML 1755 kb)
